# The Emerging Role of MicroRNAs in Bone Diseases and Their Therapeutic Potential

**DOI:** 10.3390/molecules27010211

**Published:** 2021-12-30

**Authors:** Luis Alberto Bravo Vázquez, Mariana Yunuen Moreno Becerril, Erick Octavio Mora Hernández, Gabriela García de León Carmona, María Emilia Aguirre Padilla, Samik Chakraborty, Anindya Bandyopadhyay, Sujay Paul

**Affiliations:** 1 Tecnologico de Monterrey, School of Engineering and Sciences, Campus Querétaro, Av. Epigmenio González, No. 500 Fracc. San Pablo, Querétaro 76130, Mexico; a01208914@tec.mx (L.A.B.V.); a01209117@itesm.mx (M.Y.M.B.); a01704348@itesm.mx (G.G.d.L.C.); a00821939@itesm.mx (M.E.A.P.); 2 Tecnologico de Monterrey, School of Engineering and Sciences, Campus Mexico City, Calle del Puente, No. 222 Col. Ejidos de Huipulco, Tlalpan, Mexico City 14380, Mexico; a01656501@itesm.mx; 3 Division of Nephrology, Boston Children’s Hospital, Harvard Medical School, Boston, MA 02115, USA; samik8981@gmail.com; 4 International Rice Research Institute, Manila 4031, Philippines; Anindya.B@ril.com; 5 Reliance Industries Ltd., Navi Mumbai 400701, India

**Keywords:** microRNAs, bone diseases, metastasis, biomarker, gene regulation, therapeutics

## Abstract

MicroRNAs (miRNAs) are a class of small (20–24 nucleotides), highly conserved, non-coding RNA molecules whose main function is the post-transcriptional regulation of gene expression through sequence-specific manners, such as mRNA degradation or translational repression. Since these key regulatory molecules are implicated in several biological processes, their altered expression affects the preservation of cellular homeostasis and leads to the development of a wide range of pathologies. Over the last few years, relevant investigations have elucidated that miRNAs participate in different stages of bone growth and development. Moreover, the abnormal expression of these RNA molecules in bone cells and tissues has been significantly associated with the progression of numerous bone diseases, including osteoporosis, osteosarcoma, osteonecrosis and bone metastasis, among others. In fact, miRNAs regulate multiple pathological mechanisms, including altering either osteogenic or osteoblast differentiation, metastasis, osteosarcoma cell proliferation, and bone loss. Therefore, in this present review, aiming to impulse the research arena of the biological implications of miRNA transcriptome in bone diseases and to explore their potentiality as a theragnostic target, we summarize the recent findings associated with the clinical significance of miRNAs in these ailments.

## 1. Introduction

Bones are mineralized structures of connective tissue that are made up of osteoblasts, osteoclasts, osteocytes, and bone lining cells. The skeletal system represents around 15% of the body weight and owns several vital functions, such as support, locomotion, soft tissue protection, production of blood cells, as well as calcium and phosphate storage. In addition, bones are constantly resorbed by osteoclasts and formed by osteoblasts; as a matter of fact, bone homeostasis is preserved due to the equilibrium between these bone-resorbing and bone-forming mechanisms [1,2]. As observed in different studies, the alteration of the abovementioned homeostatic balance leads to the development of diverse bone diseases that aggravate mobility problems and mortality [3,4]. Unfortunately, numerous therapies designed to treat bone diseases require the oral or bolus administration of high drug doses that negatively affect other organs [4]. Consequently, the search for novel therapeutic targets for treating bone diseases with minimum side effects represents a medical challenge that must be addressed in the upcoming years.

**Figure 1:**
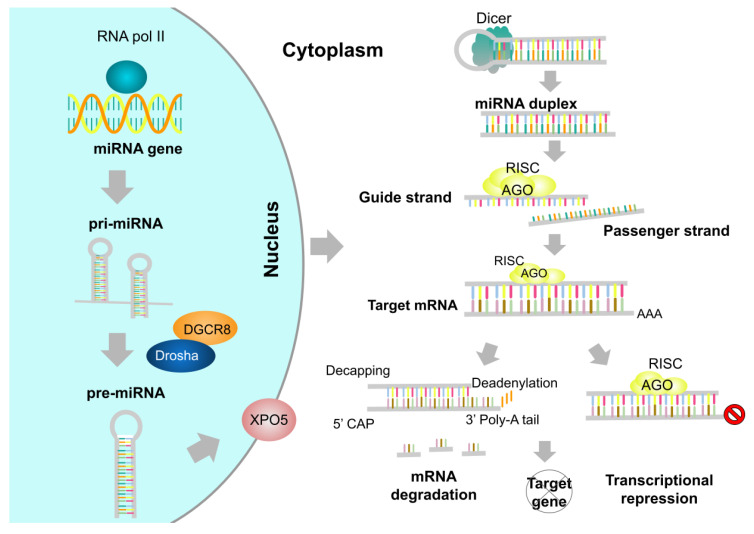
miRNA biogenesis pathway. The pri-miRNA is transcribed in the nucleus by RNA polymerase II and is subsequently converted into pre-miRNA by the Drosha complex and DGCR8. Following this, XPO5 has transported it into the cytoplasm, where the Dicer enzyme removes the hairpin loop, thus forming the miRNA duplex. Afterward, the duplex strands are separated, and the guide strand forms the RISC complex with AGO. Finally, the RISC complex binds to the respective target mRNA and either degrades or represses it.

MicroRNAs (miRNAs) are small (20–24 nucleotides), non-coding RNA molecules that can modulate gene expression in eukaryotic organisms post-transcriptionally [5]. The existence of miRNAs was first reported (1993) in the nematode *Caenorhabditis elegans* and later in other relevant organisms, including *Homo sapiens* (humans), *Mus musculus* (mice), *Arabidopsis thaliana*, and *Drosophila melanogaster* [6,7]. The biogenesis process of miRNAs begins when long primary miRNAs (pri-miRNAs) are transcribed in the nucleus by RNA polymerase II. Afterward, the Drosha complex (conformed by Drosha RNase II, DGCR8, and other associated proteins) processes these pri-miRNAs into hairpin structures known as precursor miRNAs (pre-miRNAs). Once Drosha-mediated processing has been completed, pre-miRNAs are transported to the cytoplasm by exportin-5 (XPO5), where the Dicer RNase III, with the support of Dicer-associated proteins, interacts with the pre-miRNAs to produce mature miRNA duplexes [8]. Subsequently, one of the strands of the miRNA duplex, known as the guide strand, generates the RNA-induced silencing complex (RISC) with an argonaute (AGO) protein and other important polypeptides. Finally, the RISC interacts with its complementary mRNA target and regulates its expression (Figure 1).

In the past few years, several reports have shown that the altered expression of miRNAs contributes to the development of a wide range of human pathologies, and hence these tiny molecules have a remarkable potential to be used both as biomarkers and therapeutic targets for human illnesses, such as cancer, diabetes, chronic pediatric diseases, parasitic diseases, child neuropsychiatric disorders, hair loss disorders, cardiovascular diseases, and COVID-19 [9,10,11,12,13,14,15,16]. In this context, different studies have been conducted to understand the roles of miRNAs in the development of bones and their association with the progression of bone diseases [17,18,19,20]. For instance, the most common miRNAs related to osteoblast functions during postnatal skeletal development are miR-34, miR-182, miR-199, miR-214, and miR-2861 [21]. While, relevant analyses have pointed out that miR-29b, miR-125b, miR-204, miR-211, miR-133, miR-135, miR-378, miR-2861, miR-3960, among other miRNAs, are implicated in the regulation of osteoblastic differentiation [22]. Besides, it has also been reported that dysregulation of numerous miRNAs is associated with the development of common bone diseases, including osteoporosis (e.g., miR-133a, miR-138, and miR-214), osteoarthritis (e.g., miR-34a, miR-181a, and miR-455-3p), and rheumatoid arthritis (e.g., miR-16, miR-124a, and miR-155) [23].

A number of circulating miRNAs that are present in the extracellular environment (e.g., serum, plasma, saliva, urine, and breast milk) have also drawn attention due to their high stability and possible involvement in pathophysiological conditions [24,25]. Circulating miRNAs are implicated in cell–cell communication, and since these miRNAs have the ability to affect distant tissues, they can enhance the progression and development of several diseases. For instance, circulating miRNAs secreted by cancer cells have been associated with tumorigenesis in recipient cells [26]. Furthermore, these extracellular miRNAs have excellent potential to be used as molecular diagnostic tools to identify certain diseases, such as lung cancer, colorectal cancer, and diabetes [27,28,29]. Additionally, circulating miRNAs participate in the molecular mechanisms of cell communication in the bone niche and bone metabolism, and hence they might possess a fundamental role in bone homeostasis [20]. Consequently, several relevant reports have shown that circulating miRNAs participate in the pathogenesis and progression of bone diseases, including multiple myeloma, osteoporosis, osteoarthritis, and osteosarcoma; besides, they could be used as biomarkers for these diseases [30,31,32,33].

Considering the previous information, miRNAs represent promising theragnostic targets for bone diseases that might help overcome the dose-associated side effects and the delivery challenges of conventional treatments. Thus, in this review, we address an overview of the recent crucial investigations concerning the biological and therapeutic implications of miRNAs in the pathogenesis of bone diseases. Besides, we discuss some of the most relevant concerns that should be addressed in future research for better miRNA-mediated disease management.

## 2. Osteoporosis and miRNAs

Osteoclasts and osteoblasts are body cells that resorb and synthesize the bone matrix in a synchronized manner for natural bone remodeling. However, the loss of homeostatic balance between osteogenesis and bone resorption is considered a potential factor that contributes to the development of osteoporosis [34], a degenerative disease characterized by a decrease in bone mineral density and bone tissue deterioration. Hence, this pathology results in bone fragility and a high risk of bone fractures. Interestingly, osteoporosis is more frequent in aged people and postmenopausal women [35,36]. Numerous investigators have studied the most common miRNAs related to the regulation of osteogenesis in osteoporosis, such as let-7a-5p, miR-10b, miR-221-5p, miR-203-3p, miR-590-5p, miR-1297, miR-9-5p, miR-129-5p, miR-135a-5p, miR-338-3p and miR-205 [37].

In this context, different reports have elucidated that miRNAs are regulators of proteins involved in osteoporosis progression, including glutaminase (GLS), rapamycin-insensitive companion of mammalian target of rapamycin (RICTOR), insulin-like growth factor-2 (IGF2), proto-oncogene Wnt-1 (WNT1), special AT-rich sequence-binding protein 2 (SATB2), among others [34,35,38,39,40]. Additionally, these tiny RNA molecules have been found to promote the PTEN/PI3K/AKT signaling pathway, which is associated with the differentiation and survival of osteoclasts and osteoblasts [41].

Additionally, it is worth mentioning that the hormone estrogen, which is linked with multiple diseases (e.g., osteoporosis, obesity, cancer, infertility, and endometriosis), displays a protective role on bones owing to the fact that it can inhibit bone resorption of osteoclasts [42,43]. Interestingly, estrogen could downregulate the expression of miR-21, thus increasing the expression of FasL and inducing osteoclastic apoptosis [44]. This hormone also promotes osteoprotegerin production in MG-63 cells by suppressing miR-145 expression. Since osteoprotegerin is a molecular receptor that reduces bone resorption by blocking osteoclast development, its enhanced synthesis could significantly affect bone metabolism and postmenopausal osteoporosis [45]. Moreover, estrogen is associated with the regulation of bone marrow mesenchymal stem cells (BMSCs), and the estrogen receptor α (ER-α) has a crucial role in osteoblast metabolism [43].

Studies regarding the functional implications of miRNAs on estrogen receptors and osteoporosis are still limited. However, since estrogen production is deprived in postmenopausal women due to loss of function of the ovaries [46], future research should focus on unveiling the regulatory activities of miRNAs on/by estrogen receptors during osteoporosis. Case in point, it has been reported that the let-7 miRNA family can regulate the expression of ER-α in patients with breast cancer [47]. Other miRNAs, such as miR-1, miR-9, miR-20a/b, miR-22, miR-122, miR-583, miR-874, miR-1231, are also able to regulate the expression of ER-α in breast cancer [48].

Accordingly, the roles of miRNAs on estrogen receptors during osteoporosis are yet to be elucidated. In this regard, it has been observed that anti-miR-148a generates a protective effect against ovariectomy-induced osteoporosis through the activation of PI3K/AKT signaling due to an increased expression of ER-α [49]. Besides, the circulating miRNA miR-122-5p has been proposed as a potential biomarker for osteoporosis, and one of its putative targets is ER-α [50]; nevertheless, further inquiries are required to illustrate the regulatory function of miR-122-5p on ER-α.

In 2018, Wang et al. noticed a significant downregulation of miR-144-3p in osteoporotic serum and bone tissue, altering osteoclastogenesis by targeting receptor activator of NF-κB (RANK); the authors suggest that this miRNA should be evaluated as a serum osteoporosis biomarker for therapeutic applications [51]. Furthermore, Li et al. [52] showed that miR-133a was highly upregulated in serum samples from postmenopausal osteoporotic women targeting RUNX2 [53], and the overexpression of this miRNA induced osteoclast differentiation and loss of lumbar spine bone mineral density [52].

Li et al. [41] conducted a study in which the miRNA profile was investigated in the serum and bone tissues of osteoporosis and non-osteoporosis patients. The results revealed that miR-363-3p was upregulated in the samples of osteoporosis patients, promoting osteoclastogenesis and inhibiting osteogenic differentiation. Besides, cell transfection of the myeloblastosis transcription activator (MYB) into CD14+PBMCs allowed the transcription activation of miR-363-3p, which subsequently triggered the PI3K/AKT signaling pathway via targeting PTEN, a tumor suppressor implicated in cell proliferation, adhesion, motility, and apoptosis [54]. Consequently, this process led to the stimulation of osteoporosis pathogenesis, thus indicating that miR-363-3p inhibition may represent a novel approach for osteoporosis treatment [41]. Lian et al. [55] reported that estrogen deficiency might induce the downregulation of miR-29a in osteogenic cells. As a consequence, the altered expression of miR-29a triggered the overproduction of the receptor activator of nuclear factor kappa-B ligand (RANKL) and C-X-C motif chemokine ligand 12 (CXCL12) and enhanced osteoclastic resorption and osteoporosis.

In addition, Feng et al. [35] revealed that the increased expression of miR-152 inhibited osteoblast differentiation in femoral tissues of ovariectomy-induced osteoporotic rats by targeting RICTOR, a protein associated with osteoblast activity and bone resorption. Interestingly, the repression of miR-152 promoted osteoblast differentiation and mitigated osteoporosis through the upregulation of RICTOR [35]. Moreover, Li et al. [38] demonstrated that the overexpression of miR-449b-5p prompts *SATB2* downregulation in bone marrow mesenchymal stem cells (BMSCs), which in turn aggravates osteoporosis due to the inhibition of osteogenic differentiation. Furthermore, miR-449b-5p could impede BMSCs differentiation through the reduction of RUNX2 and OCN expression and downregulating the activity of alkaline phosphatase (ALP) [38]. These results widen the knowledge of osteoporosis pathogenesis and could lead to innovative miRNA-based treatments. In another investigation, the potential roles of miR-451a in osteoporosis were analyzed in mesenchymal stem cells (MSCs) and primary osteoblasts of miR-451a knockout, as well as ovariectomized mice models. The results highlighted that the expression of bone morphogenetic protein 6 (BMP6, which is remarkably associated with osteogenesis) is negatively regulated by miR-451a. Therefore, when miR-451a was suppressed, mineralization and osteoblast differentiation were enhanced, and hence bone formation was augmented owing to the BMP6-induced activation of SMAD1/5/8 [56].

Luo et al. [57] analyzed the serum samples of osteoporosis patients and human MSCs, and they concluded that miR-579-3p plays a crucial role in osteoporosis by promoting its progression, since the NAD-dependent protein deacetylase sirtuin-1 (SIRT1, which is related to osteoblasts differentiation) is directly regulated by miR-579-3p. Therefore, when this miRNA is overexpressed, osteogenic differentiation is inhibited. Thus, these outcomes provide new fundamentals for the clinical management of osteoporosis [57]. Another study suggested that miR-200a-3p is highly expressed in the serum of osteoporosis patients, negatively regulating the expression of glutaminase (GLS), which is linked with the osteogenic differentiation of BMSCs, thus hastening osteoporosis progression. Interestingly, this process could be reversed through GLS overexpression. Overall, these findings serve as a valuable alternative for novel osteoporosis therapies [58]. 

Additionally, the impact of the long non-coding RNA (lncRNA) MSC antisense RNA 1 (MSC-AS1) on osteoporosis and its interplay with miR-140-5p were tested in the bone marrow-derived cells of mice. The results revealed that miR-140-5p directly targets the bone morphogenetic protein 2 (BMP2), which plays an essential role in osteogenesis and bone formation. Intriguingly, MSC-AS1 can modulate the expression of miR-140-5p by absorbing it, thus allowing the occurrence of the BMP2/SMAD signaling pathway, which in turn triggers osteogenic differentiation. This sponging effect of MSC-AS1 against miR-140-5p might set the basis for innovative osteoporosis therapies [59]. Zhou et al. [60] noticed that the downregulation of miR-339 in mice’s bone marrow-derived mesenchymal stem cells might allow the expression of its target gene *DLX5* and enhance osteogenic differentiation, thus promoting the improvement of osteoporosis. Moreover, another relevant report illustrated that miR-140-3p had been upregulated, and the guanine nucleotide exchange factor MCF2L had been downregulated in osteoporosis patients. Conversely, overexpression of miR-140-3p in MC3T3-E1 preosteoblasts reduced MCF2L expression, restraining the differentiation of preosteoblasts and prompting their apoptosis [61]. This evidence established that *MCF2L* is a latent gene correlated with osteoporosis development; hence the induction of miR-140-3p expression could be relevant for treating this bone disease; notwithstanding, more analyses are required to unveil the impact of miR-140-3p/MCF2L on osteoporosis [61].

Furthermore, Mi et al. [62] reported that miR-194-5p is upregulated in the plasma samples from osteoporosis patients, which is inversely associated with the degree of bone formation in these affected individuals. Subsequently, it was detected that this miRNA inhibits osteoblast differentiation and diminishes bone mass in vivo and impedes osteogenic differentiation in vitro. Besides, it was found that the target of miR-194-5p is the Wnt-5a protein (WNT5A), whose main function is related to the induction of osteogenic differentiation through the Wnt5a/β-catenin signaling pathway. Accordingly, the increased expression of miR-194-5p during osteoporosis affects osteogenic differentiation and aggravates bone loss via targeting Wnt5a [62]. In addition, it has been stated that frizzled-4 (FZD4), a protein associated with osteogenic differentiation, is a potential target of miR-1286. Since this miRNA is upregulated in the serum of osteoporosis patients, it negatively controls the FZD4 expression, thus impeding the osteogenic differentiation of human BMSCs and promoting the progression of osteoporosis [63]. 

Crucially, in an analogous study, miR-483-5p was upregulated, and IGF2 downregulated in bone tissues and serum samples isolated from osteoporosis patients. Subsequently, CD14+ peripheral blood mononuclear cells were isolated, and these cells were transfected with a miR-483-5p mimic, whose target is IGF2; the results elucidated that miR-483-5p is significantly associated with the pathogenesis of osteoporosis, since it promotes osteoclasts differentiation via silencing IGF2 [34]. In a following inquiry, the implications of miRNAs in osteoporosis were studied in ovariectomy rats subjected to osteo-anabolic (with teriparatide) and anti-resorptive (with zoledronate) treatments. As a result, the expression of 11 miRNAs was affected by these therapeutic procedures [40]. In fact, one of the most representative upregulated miRNAs during the bone loss induced by the ovariectomy was miR-203a; however, its expression became reduced after the anti-osteoporotic treatments. It is worth mentioning that these changes in miR-203a expression also affected the activity of genes linked with bone formation, such as *DLX5*, which is the direct target of miR-203a. In summary, the use of anti-osteoporotic treatments (teriparatide and zoledronate) represent a prominent source for minimally invasive strategies for postmenopausal osteoporosis treatment [40].

**Figure 2:**
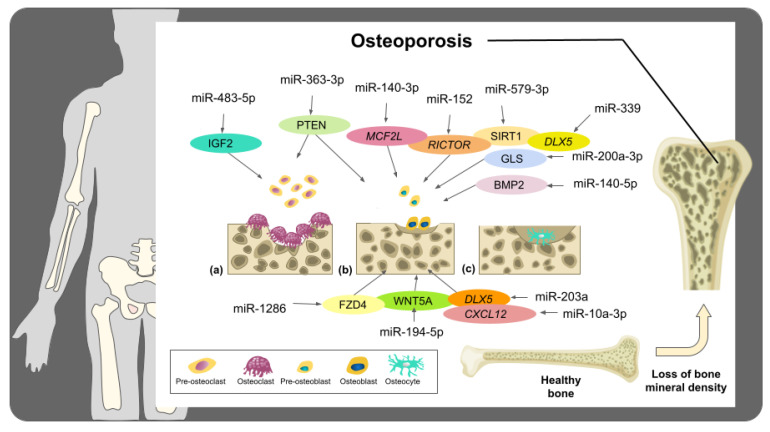
Schematic representation of some of the important miRNAs associated with osteoporosis progression. These miRNAs have been described as relevant regulators of targets associated with bone mineralization, osteogenic differentiation, and osteoclast differentiation. When bone resorption increases (**a**) and osteogenesis is restrained (**b**), osteocyte lacunae decrease (**c**), which subsequently drives to the loss of bone mineral density and the occurrence of osteoporosis.

Interesting findings clarify that the restraint of miR-10a-3p caused by the flavonoid Kaempferol in BMSCs triggers the expression of its target CXCL12 and promotes osteogenic differentiation (Figure 2). Since the Kaempferol treatment in rats with ovariectomized-induced osteoporosis boosted bone density, these findings denote a novel alternative for the prevention and handling of osteoporosis by promoting osteogenic differentiation via targeting miR-10a-3p [64].

According to the outcomes obtained from the aforesaid studies, miRNAs have remarkable potential in the clinical procedures involved in the prognosis and treatment of osteoporosis. Nonetheless, more studies are required to determine which miRNAs are reliable biomarkers for osteoporosis since, in several studies, sample sizes were reduced, and volunteers’ dietary supplements were not controlled; as a consequence, these factors might contribute to obtaining contrasting results [65]. On the other hand, to the best of our knowledge, none of the osteoporosis-related miRNAs studied so far have been entered into human clinical trials, and hence there is a wide opportunity to continue studying those miRNAs that have shown promising results in biological models of osteoporosis to find potential candidates to develop miRNA-based drugs for this disease.

## 3. Osteosarcoma and miRNAs

Osteosarcoma is the primary and most common type of bone cancer in young adults and children, mostly occurring in long tubular bones causing serious pain, swelling, and joint dysfunction [66]. The etiology and genetics of this disease are very complex due to a large variety of mutations responsible for triggering the syndromes that predispose osteosarcoma [67]. Additionally, the genotype of osteosarcoma is found to be constantly changing [68]. In this context, different studies have been focused on elucidating the potential roles of miRNAs in diverse aspects of osteosarcoma, including diagnostic and therapeutic approaches. For instance, previous reports have elucidated that many miRNAs, including miR-34 family, miR140, miR-92a, miR-99b, miR-132, miR-21, miR-143, miR-33b, miR-133b, miR-221, miR-17, miR-143, miR-144, miR-27a, miR-20a, amongst others, are implicated in osteosarcoma pathology [69,70].

To begin with, hypermethylation of the CpG island located upstream of the miR-300 locus is one of the most important causes of tumorigenesis in human osteosarcoma cells. Accordingly, the repressed expression of miR-300 allows CUL4B protein to express abundantly, which in turn increases CRL4B^DCAF13^ E3 ligase activity and promotes the degradation of PTEN, a crucial tumor suppressor [71]. Under this premise, ectopic expression of miR-300 in osteosarcoma cells affected the stability of CRL4B^DCAF13^ E3 ligase and diminished the ubiquitination of PTEN. Similar results were obtained when treating osteosarcoma cells with the DNA methylation inhibitor 5-AZA-2′-deoxycytidine (AZA). These outcomes reveal a novel molecular mechanism to design therapeutic strategies for osteosarcoma [71]. A further investigation revealed that miR-93 is upregulated in osteosarcoma tissues and cells (i.e., Saos-2, U2OS, SW1353, and MG63) and that it has a fundamental implication in osteosarcoma development since it inhibits the expression of P21, a protein that modulates the cell cycle during the G1 checkpoint. Afterward, researchers transfected osteosarcoma cells with a miR-93 inhibitor and observed that the expression of miR-93 was lessened; moreover, the proliferation of both U2OS and MG63 osteosarcoma cells was inhibited [72]. Therefore, the inhibition of miR-93 could represent a viable strategy for osteosarcoma treatments.

Xu et al. [73] unveiled that the expression of miR-411 was significantly higher in serum samples obtained from osteosarcoma patients than that of healthy subjects. Additionally, miR-411 was found to be upregulated in osteosarcoma tissues. Subsequently, researchers observed that the overexpression of miR-411 stimulates both osteosarcoma cell migration and proliferation via reducing the expression of metastasis suppressor protein 1 (MTSS1). Therefore, the inhibition of miR-411 could be a reliable method to treat osteosarcoma progression. Previous investigations have supported the fact that miR-1284 is linked with different types of cancer [74,75]. To explore the role of this regulatory molecule in osteosarcoma, tumor tissues were examined, and the outcomes of this analysis indicated that the expression of miR-1284 was lower in tumor tissues when compared to adjacent healthy tissues. Moreover, the expression of HMGB1 was considerably higher in tumor tissues, thus revealing that the expression of this protein might be negatively correlated with the occurrence of miR-1284. The main functions of HMGB1 are related to DNA-associated processes, including replication, translation, and repair; however, its role within osteosarcoma development may be related to the inhibition of osteosarcoma cell migration and proliferation [76]. Additionally, Hua et al. [77] noticed that the survival rate of osteosarcoma patients with an elevated expression of blood let-7a (negatively regulates the E2F2, a transcription factor involved in diverse cancer pathways) was higher than that of patients with inferior expression of let-7a, and hence this miRNA could be a potent biomarker for osteosarcoma.

Remarkably, the interaction of miRNAs with lncRNAs is an interesting phenomenon that is gaining more and more attention in cancer research [78]. Under this premise, Zhu and colleagues [79] found that the lncRNA small nucleolar RNA host gene 16 (SNHG16) was highly expressed in osteosarcoma cell lines and tissues. Conversely, researchers observed that the downregulation of SNHG16 enhances osteosarcoma cell proliferation. The molecular basis of this functional implication lies in the fact that SNHG16 acts as an endogenous sponge of miR-205; accordingly, this sponging effect promotes the upregulation of ZEB1, a transcription factor correlated with tumor invasion and metastasis [80]. Another study carried out in osteosarcoma tissues, and cell lines demonstrated a significant downregulation of miR-1301. However, the overexpression of this miRNA impeded cell proliferation, invasion, and migration via targeting BCL9 (which is involved in the proliferation of different types of cancer). Therefore, miR-1301 might be a promising therapeutic target for osteosarcoma [81]. Huang et al. [82] demonstrated that miR-487a, miR-493-5p, miR-501-3p, and miR-502-5p were significantly upregulated among osteosarcoma patients. Nevertheless, since this study possesses several limitations (e.g., small sample size and no inquiry regarding the pathologic effects of these miRNAs), more investigations are needed to unveil the roles of the aforesaid miRNAs in osteosarcoma [82].

**Figure 3:**
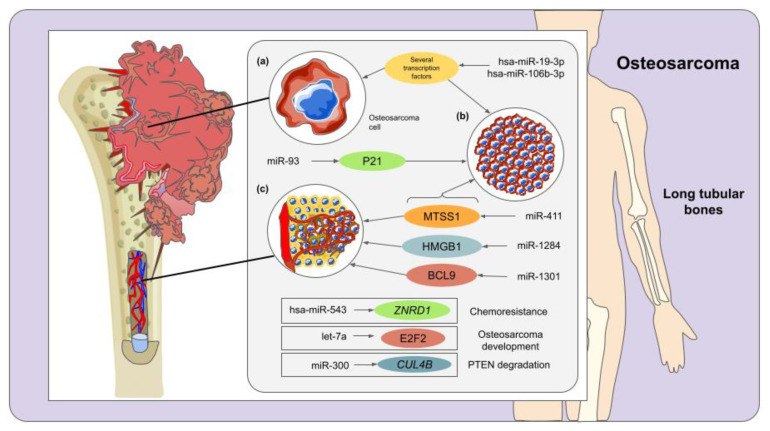
Crucial miRNAs involved in osteosarcoma development and progression. Carcinogenesis (**a**), cell proliferation (**b**), and cell invasion and migration (**c**) have been identified as the most illustrative biological implications for miRNAs in osteosarcoma disease. Some of these miRNAs exhibit more than one role in the main stages of osteosarcoma pathology. Furthermore, these regulatory molecules enclose a remarkable potential as biomarkers for osteosarcoma.

In a relevant study, a meta-analysis supported by the existing literature and bioinformatic tools revealed that 4 miRNAs (hsa-miR-106, hsa-miR-17, hsa-miR-181, and hsa-miR-19) are upregulated, and 8 (hsa-miR-126, hsa-miR-127, hsa-miR-133, hsa-miR-150, hsa-miR-335, hsa-miR-376, hsa-miR-409, and hsa-miR-451) are downregulated in osteosarcoma. Intriguingly, the overexpressed miRNAs typically target transcription factors, while the targets of the downregulated miRNAs were found to participate in the secretion of tumor necrosis factor and phosphorus metabolism [83]. Accordingly, hsa-miR-19-3p and hsa-miR106b-3p, as well as the transcription factor SIX3 (which is a promising suppressor in human lung cancer), were predicted as relevant biomarkers and potential therapeutic targets for osteosarcoma. Nevertheless, since all these observations were achieved through bioinformatic analyses, experimental validation is required to confirm the functional implications of the miRNAs examined in this study [83]. Similarly, a bioinformatic analysis was performed with the aim of identifying differentially expressed miRNAs and associated genes among osteosarcoma patients with chemoresistance revealed a list of 5 differentially expressed miRNAs and 668 differentially expressed genes. Among them, hsa-miR-543, as well as *ZNRD1*, *GPR68*, *CAT*, *FUT3*, *ANPEP*, and *CDK1* genes, were proposed as crucial players in osteosarcoma chemoresistance, and hence they could represent potential therapeutic targets for osteosarcoma [84] (Figure 3).

In summary, many miRNAs have noteworthy potential for the clinical management and diagnosis of osteosarcoma. However, to date, clinical trials of miRNA-based therapeutics for this disease have not yet been performed and, even though the outcomes observed both in vivo and in vitro are encouraging, before an miRNA-based therapy approach for patients with osteosarcoma could be explored, thorough toxicity studies and preclinical safety must be assessed extensively [85]. In addition, to validate the use of miRNAs as biomarkers for osteosarcoma, long-term, controlled studies with large sample sizes are needed [85]. Under this premise, forthcoming studies should be focused on designing miRNA-based drugs for osteosarcoma along with efficient delivery systems to examine the therapeutic effects and safety of these tiny RNA molecules in animal models prior to entering clinical trials.

## 4. Osteonecrosis and miRNAs

Bones are constantly maintained and remodeled by a delicate balance of osteoblasts and osteoclasts; nonetheless, the alteration of this equilibrium may trigger decreased rates of osteogenesis and increased rates of adipogenesis that lead to a bone disease called osteonecrosis [86,87]. Osteonecrosis is characterized by the necrosis of bone tissue and bone marrow due to a failure in blood circulation that results in the collapse of the affected subchondral bone [88]. Osteonecrosis of the femoral head (ONFH) is one of the most common types of osteonecrosis that occurs predominantly in male individuals between 30 and 50 years of age [89]. ONFH pathogenesis can be associated with either traumatic or non-traumatic circumstances [90]. Non-traumatic ONFH can be triggered by long-term high doses of glucocorticoids (GC), alcoholism, chemotherapy, exposure to cytotoxic agents, or genetic factors; nevertheless, there are several knowledge gaps concerning the etiology of this disorder [91,92]. On the other hand, traumatic ONFH is induced by physical traumas such as femoral neck fracture, which affects normal blood circulation [93].

One of the main consequences of ONFH is the collapse of the femoral head due to a constant imbalance between bone formation and bone resorption that decreases the strength of the bone structure. In this context, Li et al. [94] analyzed the profile of bone tissue samples obtained from the collapsed, non-collapsed, and normal areas of osteonecrosis femoral heads. Intriguingly, they found that hsa-miR-195-5p was significantly downregulated only in the collapsed area, which might lead to a disruption of dissemination of normal osteoblasts and accelerated cell apoptosis, causing weakness in the femoral head and, consequently, its collapse. In addition, 157 genes were predicted as potential targets of has-miR-195-5p. Nevertheless, further research is needed to experimentally validate these targets and establish novel miRNA-based treatments to prevent the collapse of the femoral head.

It has been reported that glucocorticoids (GC) have an antagonistic role in MSCs since they can induce intramedullary adipogenesis, vascular endothelial injury, intramedullary hypertension, and microvascular thrombosis that causes failure in the normal blood supply to the femoral head, leading to the steroid-induced osteonecrosis of the femoral head (SONFH) [95]. Considering this, MSCs of patients with non-traumatic ONFH were analyzed to identify miRNAs related to SONFH, and among 22 studied miRNAs, only 6 were found associated with osteogenic and adipogenic differentiation, which are processes involved in SONFH [87]. Moreover, it was detected that hsa-miR-601, hsa-miR-452-3p, hsa-miR-647, hsa-miR-516b-5p, and hsa-miR-127-5p were significantly upregulated, while hsa-miR-122-3p was downregulated in SONFH patients and during adipogenic differentiation; notwithstanding, these expression patterns were reversed during osteogenic differentiation. As observed, these miRNAs could be potential biomarkers and clinical targets for SONFH; however, the identification of their target genes would be of great relevance to understand their precise role in this disease [87]. On the other hand, Xie et al. [88] reported that the expression of miR-181d was upregulated in the bone marrow of SONFH patients, negatively regulating its target *SMAD3*. Interestingly, the silencing of this particular miRNA inhibits the differentiation of human BMSCs into osteoblasts. Additionally, cell transfection of a miR-181d inhibitor enhanced the expression of *SMAD3*, thus suggesting an opportunity area for potential SONFH treatments.

In another study, miR-217 was found to be inversely associated with *DKK1* expression, a gene linked with bone metabolism. In this regard, it was elucidated that in the MSCs (isolated from femur bone marrow of ONFH patients), *DKK1* was overexpressed due to the downregulation of miR-217, resulting in the inhibition of cell proliferation and osteoblastic differentiation [96]. Furthermore, researchers observed that the artificial overexpression of miR-217 could promote osteogenic differentiation and repair of femoral head bone tissue via suppressing *DKK1* [96]. In 2019, Wang et al. [97] designed an miRNA-based approach to regulate osteoblast and osteoclast activities using the AAV-anti-miR-214 to prevent the collapse of the femoral head in ONFH patients. They found a promising therapeutic use of miR-214 due to its dual regulatory activity, where its upregulation can cause the inhibition of osteoblastic differentiation targeting the transcription factor 4 (ATF4) and promote osteoclastic differentiation by targeting phosphatase and tensin homolog (PTEN). Afterward, AAV-anti-miR-214 was applied to rodent models, improving bone formation; likewise, a reduction in bone resorption was perceived due to increased PTEN expression. These results suggest that the therapeutic inhibition of miR-214 could prevent femoral head collapse and postpone ONFH progression at the early stages of the disease [97].

Furthermore, Xu et al. [89] revealed that miR-186-5p is strongly expressed in human MSCs from individuals with non-traumatic osteonecrosis. Researchers reported that this miRNA targeted the C-X-C motif ligand 13 (CXCL13), a chemokine that regulates cell proliferation and stimulates osteoblast differentiation. Additionally, CXCL13 mRNA was negatively correlated with miR-186-5p expression in bone marrow samples from non-traumatic osteonecrosis patients, directly affecting cell viability and osteoblastic differentiation. Furthermore, miR-186-5p overexpression inhibited the activation of the AKT/ERK signaling pathway, thus altering MSC’s growth [89]. An in vivo assay performed by Yin et al. [98] on Sprague–Dawley rats to prevent ONFH by targeting Wnt-11 (a positive regulator that has a key function in carcinogenesis) through miR-410 overexpression revealed that this miRNA mediates the downregulation of Wnt-11-enhanced bone mineral density, as well as bone volume fraction; this inverse relationship increased osteoblast production while lowering osteoclast synthesis, and therefore mitigated ONFH.

**Figure 4:**
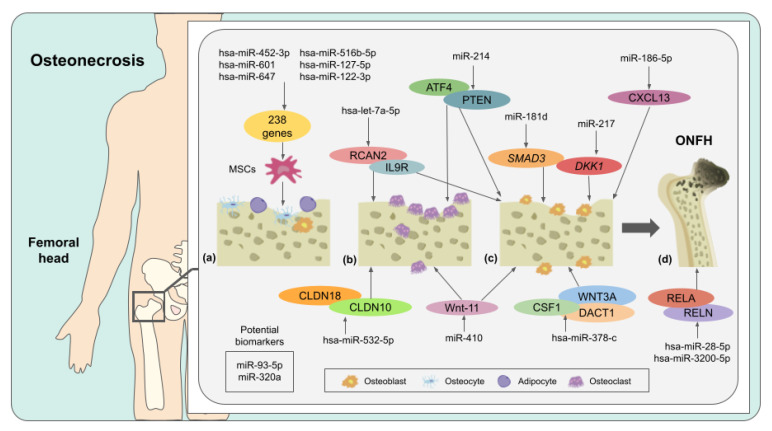
miRNAs and their respective targets (and functions) associated with the pathology of ONFH. Osteogenic and adipogenic differentiation (**a**), osteoclast proliferation (**b**), osteoblast proliferation (**c**), and cartilage degeneration (**d**) are the most representative roles of these miRNAs in ONFH. Remarkably, a number of these miRNAs represent potential biomarkers that might be used to diagnose this disease.

Hao et al. [99] identified 24 differentially-expressed miRNAs associated with 457 mRNAs in blood samples of ONFH individuals; some of the most representative upregulated miRNAs were hsa-miR-378c, hsa-miR-3200-3p, hsa-miR-28-5p, hsa-let-7a-5p, hsa-miR-3200-5p, and hsa-miR-532-5p. Moreover, it was observed that hsa-miR-378-c could downregulate WNT3A, DACT1, and CSF1, suggesting its possible role in regulating angiogenesis and bone remodeling. Furthermore, hsa-miR-3200-5p and hsa-miR-28-5p suppressed the expression of RELN and RELA, respectively, indicating their role in ONFH and cartilage degradation. Likewise, it was suggested that hsa-let-7a-5p could have a crucial role in ONFH progression due to the fact that this miRNA targets RCAN2 and IL9R, which are proteins involved in bone development and hematopoietic cell lineage, correspondingly. Finally, it was demonstrated that hsa-miR-532-5p could hinder the expression of CLDN18 and CLDN10 (Figure 4), which might be implicated in ONFH bone loss [99].

Later, fourteen potent miRNA biomarkers for traumatic ONFH were computationally identified, and three of their main target genes (*GSK3β*, *CCND1*, and *NFκB1*) were found to be associated with several signaling pathways, such as Wnt PI3K, Akt, AMPK and Hippo, which are strongly correlated with ONFH development [86]. In addition, a qRT-PCR assay revealed that miR-93-5p and miR-320a were significantly upregulated in serum from ONFH patients, thus suggesting that these miRNAs could be considered as principal biomarkers for early diagnosis and therapy of this bone condition [86]. Recently, in 2021, Yue et al. [100] demonstrated that the flavonoid icariin could lower the expression of miR-335, a possible regulator of steroid-induced ONFH, in rat bone microvascular endothelial cells. Further bioinformatics analyses revealed that miR-335 could target 113 genes, among which 101 had a regulatory effect enhanced by icariin. Nevertheless, the authors indicated that more studies are needed to comprehend the mechanism of this active compound to prevent ONFH.

Undoubtedly, miRNAs have an emerging role as both biomarkers and therapeutic targets for osteonecrosis. Nevertheless, large-cohort studies in different populations are required to validate their clinical use and understand their expression patterns along with the progression of this disease. Additionally, given that many of the patients treated with corticosteroids also present systemic inflammatory diseases, future research should consider samples from individuals with such diseases and treatment [101]. Regarding the development of miRNA-based drugs for osteonecrosis, more research utilizing tissue-specific knockout biological models should be conducted, and specialized delivery systems of miRNA mimics or inhibitors must be designed [101].

## 5. Bone Metastasis and miRNAs

Metastasis of bone tissue is a multistep process that occurs in the late stages of tumor development, and it depends on the properties of the tumor cells and the bone microenvironment. Carcinoma cells spread locally into circulatory and lymphatic systems to become circulating tumor cells. Thereafter, they invade and proliferate within distant organs to generate new tumors [102,103]. Both tumor and bone cells secrete factors that interact in a process known as the “vicious cycle” of bone metastasis. Tumor cells first release parathyroid hormone-related proteins (PTHrPs), which cause osteoblasts to secrete RANKL, thus stimulating monocyte maturation into osteoclasts. Once osteoclasts are activated, bone matrix degradation is enhanced by releasing important growth factors such as calcium (Ca^2+^) and transforming growth factor-beta (TGF-β). Consequently, these compounds bind to cancer cells and induce the synthesis of metastasis-promoting factors (e.g., PTHrPs, Jagged1, among others), triggering a more threatening tumor phenotype as well as bone cancer outgrowth [104,105]. As metastatic bone damage progresses, there is a higher risk of developing skeletal-related events (SREs) such as pathological fractures, hypercalcemia, spinal cord compressions, and myelosuppression. All these conditions are associated with pain, significant morbidity, and reduced patient survival [106,107]. 

### 5.1. Prostate Cancer Bone Metastasis

In recent years, the research interest in elucidating the role of miRNAs in numerous stages of bone metastasis formation and progression has augmented [108]. In this context, Colden et al. [109] unveiled that miR-466 is poorly expressed in different prostate cancer cell lines and clinical tissues. Subsequently, its artificial overexpression in prostate cancer cell lines promoted apoptosis and cell cycle arrest, inhibiting migration, proliferation, and invasion due to the downregulation of RUNX2 and several downstream targets of RUNX2 (e.g., ANGPTs, MMP11, FAK, osteopontin, osteocalcin, and vimentin) that are linked with prostate cancer bone metastasis. Thus, the restoration of miR-466 expression could be a novel therapy against bone metastasis [109]. Another relevant inquiry buttressed that upregulation of miR-19a-3p represses migration, invasion, and bone metastasis of prostate cancer cells by attenuating the expression of *SMAD2* and *SMAD4* (responsible for activating the TGF-β signaling pathway), associated with bone metastasis in prostate cancer [110].

Huang et al. [111] demonstrated that the upregulation of miR-582-3p and miR-582-5p inhibited bone metastasis in mouse models in vivo and repressed migration and invasion of human prostate cancer cells in vitro. Specifically, researchers found that these miRNAs inactivated the TGF- β signaling pathway (an oncogenic pathway in later cancer stages) via targeting several important factors, including SMAD2, SMAD4, and TGFBR1 (for miR-582-3p); and SMAD2, TGFBR1, as well as TGFBR2 (for miR-582-5p). TGFBR1 and TGFBR2 are proteins that phosphorylate members of the SMAD family (SMAD-2 and SMAD-3) and induce the formation of a complex with the SMAD-4 member; subsequently, this SMAD complex is translocated to the nucleus and regulates the transcription of TGF- β target genes [112]. However, further research into the simultaneous disruption of these proteins in cancer cells is required [111].

In addition, Voss et al. [113] noticed that miR-96 could positively regulate the in vitro expression of two cell-adhesion proteins, E-Cadherin and EpCAM, in prostate cancer cell line DU145. E-cadherin regulates epithelial morphogenesis and differentiation by acting as a calcium-dependent cell-cell adhesion protein [114], while EpCAM is an epithelial phenotypic marker [115]. It was also reported that in osteoblast conditioned medium, upregulated miR-96 DU145 cells displayed an enhanced adherence to osteoblast monolayers. Interestingly, authors suggest that this increased cell–cell interaction might trigger prostate cancer cells’ metastasis within the bone microenvironment and promote tumor development [113].

### 5.2. Breast Cancer Bone Metastasis

Experiments on fractured bone samples from patients with osteolytic bone metastasis of breast cancer revealed that miR-214-3p is upregulated in these tissues. Moreover, it was observed that the ablation of this miRNA in xenograft nude mice could prevent the evolution of osteolytic bone metastasis [116]. Subsequently, an in vitro experiment elucidated that the increased expression of miR-214-3p can drive osteoclastic bone resorption since this miRNA can negatively regulate TNF receptor-associated factor 3 (*TRAF3*), a gene linked with osteoclast formation. Accordingly, the induced restraint of miR-214-3p might be a feasible option for treating osteolytic bone metastasis in patients with breast cancer [116]. Cai et al. [117] illustrated that miR-124 is lowly expressed in strong invasive breast cancer cell lines, particularly in MDA-MB-231 (a cell line with high bone-metastatic characteristics). Besides, an in vivo assay carried out in Balb/c nude mice proved that both breast cancer cell survival in the bone microenvironment and cancer bone colonization could be inhibited by the injection of cells expressing miR-124. Further analysis disclosed that miR-124 negatively regulates IL-11, a pro-tumorigenic cytokine that activates the GP130-Janus kinase signaling pathway promoting an anti-neoplastic role in the progression of breast cancer to bone metastasis [117].

### 5.3. Lung Cancer Bone Metastasis

Xu et al. [118] described that miR-139-5p inhibits the expression of NOTCH1, a receptor that has been previously associated with this miRNA in colorectal cancer that enhances G1-S transition in the cell cycle and promotes tumor growth [119]. To sustain this hypothesis, an in vitro experiment was performed exposing MSCs to a conditioned medium of lung cancer cell lines A549 and L9981. Indeed, miR-139-5p expression was suppressed while NOTCH1 was upregulated. Additionally, the expression of miR-139-5p in serum from lung cancer patients with lytic bone metastasis was found to be lower than in individuals with metastasis in other tissues. Authors suggest that serum miR-139-5p could be a biomarker for lung cancer bone metastasis [118].

### 5.4. Other Bone Metastasis-Related Mechanisms

Wen et al. [120] demonstrated that the artificial overexpression of miR-34a in osteosarcoma MG-63 cells could inhibit cell invasion and diminish metastasis, as well as promote apoptosis and arrest cells in G0/G1 stages. These authors also suggest that this miRNA downregulates the expression of two major proto-oncogenes: C-IAP2 (probably via the NF-κB pathway) and anti-apoptotic B-cell lymphoma-2 (Bcl-2). Moreover, in the same research, an in vivo experiment in nude mice revealed that the overexpression of miR-34a could significantly inhibit the growth of osteosarcoma in animal skin.

Later, a study elucidated that overexpressed hsa-miR-940 in human MSCs could promote osteogenic differentiation in vitro via targeting ARHGAP1 (a suppressor of the RhoA/ROCK pathway) and FAM134A (a transmembrane protein whose osteogenic role remains undefined). In this same study, an in vivo experiment was performed implanting MDA-MB-231 cells with upregulated miR-940 on mice calvaria. Intriguingly, this assay induced the development of tumors that had extensive osteoblastic lesions due to the osteogenic differentiation of host mesenchymal cells. In addition, the protein levels of ARHGAP1 and FAM134A within the host cells were suppressed. Researchers concluded that this miRNA could trigger osteoblastic-type bone metastasis and microenvironment-dependent lesions [121].

### 5.5. Bone Metastasis and Exosomal miRNAs

To mediate intercellular communication, exosomes act as carriers of several miRNAs. Indeed, the concentration of exosomes in cancer cells is higher than in normal cells, allowing them to play an active role in metastasis [122]. In this concern, various investigations stated that exosomal miRNAs might be promising biomarkers for bone metastasis development. According to the study by Zhang et al. [123], BMSC-derived exosomes that transport miR-193a-3p, miR-210-3p, and miR-5100 can enhance lung cancer metastasis by promoting STAT3 signaling-induced epithelial-mesenchymal transition (EMT). In 2019, a group of researchers reported that the exosomal miR-20a-5p from breast cancer cells could stimulate osteoclast proliferation and differentiation by targeting SRC kinase signaling inhibitor 1 (SRCIN1), a kinase involved in cell migration [124].

Moreover, in non-small cell lung cancer (NSCLC) serum samples, three additional exosomal miRNAs were found to regulate bone metastatic proliferation via Wnt/ β-catenin signaling pathway: miR-328-3p and miR-423-3p as activators, and miR-574-5p as a suppressor [125]. Yuan et al. [126] revealed that exosomal translocation of miR-21 from breast cancer cells to osteoclasts promoted the establishment of a pre-metastatic niche by modulating the protein levels of programmed cell death 4 (PDCD4). A recent study published in 2021 described that the exosomal transport of miR-19a and integrin-binding sialoprotein (IBSP) might trigger the molecular mechanism of metastasis in osteoclasts and estrogen receptor-positive (ER^+^) breast cancer and induce the activation of the vicious cycle [127].

### 5.6. Clinical Applications of miRNAs in Bone Metastasis

As observed throughout the previous text, miRNAs hold an outstanding potential for developing innovative diagnostic protocols and clinical treatments for bone metastasis. Nevertheless, the development of efficient delivery methods to silence metastasis oncogenes represents a major issue that has slackened the advancement of miRNA-based therapeutics in oncology. Moreover, a number of small interfering RNA molecules occasionally trigger an immune response activating the toll-like receptor (TLR) pathway [128]. Accordingly, the use of miRNAs as therapeutic drugs for bone metastasis faces different challenges that should be addressed in upcoming studies. Despite the above, it has been suggested that MRX34 (a miR-34 mimic currently in clinical trials for the treatment of liver cancer) could protect against induced osteolytic disease in patients with metastatic breast cancer [128].

## 6. Other Bone Diseases and miRNAs

### 6.1. Atrophic Non-Union

Atrophic non-union is the lack of bone healing and repair process when a serious complication of fracture occurs. When atrophic non-union occurs, osteoblast differentiation and osteogenesis processes seem to be inhibited, reducing the possibility of normal fracture healing [129]. This condition often involves chronic pain and functional impairment. Multiple causes can be attributed to atrophic non-union; for instance, blood supply may play a crucial role in the process. In addition, insufficient osteogenesis may trigger atrophic non-union due to the fact that there is a lack of bony callus formation [129]. Likewise, diverse mechanical factors (e.g., degree of immobilization), biological factors (e.g., vascular supply and degree of bone loss), and host factors (e.g., age, gender, and smoking) might affect the occurrence and development of this kind of bone disease [130].

In 2015, Waki et al. [131] analyzed post-fracture tissues isolated from rat models of atrophic non-union. As a result, it was detected that the upregulated miRNAs: miR-31a-3p, miR-31a-5p, miR-146a-5p, miR-146b-5p, and miR-223-3p could be associated with the pathogenesis of atrophic non-union. However, the regulatory mechanisms of these miRNAs were not unveiled during this investigation, and hence it is unclear whether they participate in the processes that aggravate non-unions or if they are involved in an adaptative response caused by the periosteal cauterizations that were performed in the femora of rats to produce non-unions and healing fractures. Moreover, during another study, miR-221, miR-149*, miR-628-3p, and miR-654-5p were found upregulated, while let-7b*, miR-220b, miR-513a-3p, miR-551a, miR-576-5p, miR-1236, and kshv-miR-K12-6-5p were downregulated at the fractured sites of patients with atrophic non-union. Remarkably, researchers discovered that, among the aforesaid miRNAs, miR-628-3p might inhibit osteoblast differentiation via targeting RUNX2, thus showing a potential therapeutic miRNA target for the non-atrophic union that should be studied in the following years [129].

An experiment in primary human BMSCs revealed that the transcription factor PPARγ could bind to the promoter region of miR-381, triggering its expression, depriving osteogenic differentiation during non-atrophic union by targeting *WNT5A* and *FZD3*, and regulating the β-catenin nucleus translocation. Consequently, miR-381 may repress the Wnt signaling pathway. The anti-osteogenic role of miR-381 was also confirmed in femur fracture rat models, which implies that miR-381 represents a potential therapeutic target for non-atrophic union [132]. Recently (in 2020) Xie et al. [133] demonstrated that the expression of miR-1323 is significantly upregulated in human atrophic non-union fractures; in addition, researchers detected that this miRNA regulates BMP4 and SMAD4, two important proteins associated with osteoblast differentiation. Besides, miR-381 mediated repression of the aforementioned polypeptides inhibited the differentiation of MSCs through the regulation of the nucleus translocation of TAZ. These observations were strengthened in vivo when rat models of femoral fracture were treated with an miR-1323 antagomir that promoted the healing of the fractures. As observed, the inhibition of miR-1323 may represent a novel treatment for atrophic non-union [133].

In a subsequent investigation, the miRNA expression profiles of tissues obtained from atrophic non-union patients and samples of individuals with healed fractures were compared. Consistently, it was detected that nine miRNAs were significantly upregulated in the atrophic non-union tissues (i.e., hsa-miR-149*, hsa-miR-221, hsa-miR-628-3p, hsa-miR-654-5p, hsa-miRPlus-E1114, hsa-miRPlus-E1285, hsa-miRPlus-E1238, hsa-miRPlus-E1101, and hsa-miRPlus-C1115). On the other hand, 9 miRNAs, including hsa-let-7b^∗^, hsa-miR-220b, hsa-miR-513a-3p, and hsa-miR-551a, to name a few, were identified to be downregulated in atrophic non-union samples [134]. Further analysis performed in transfected human BMSCs elucidated that hsa-miR-149*, hsa-miR-221, and hsa-miR-654-5p can target the *ALPL*, *PDGFA*, and *BMP2* genes, respectively. Since these genes are involved in osteogenesis, their miRNA-based repression may contribute to the development of atrophic non-union [134].

### 6.2. Osteogenesis Imperfecta

Osteogenesis imperfecta is a set of inherited disorders characterized by brittle bones that fracture easily. This pathological condition can be incited by genetic defects and alterations in genes, such as *COL1A1*, *COL5A3*, *COL4A2*, and *COL1A2*, disturbing collagen production in the body and weakening the bones. In severe cases, a formidable number of fractures can occur [135,136]. Numerous investigations have shown that miRNAs can be expressed at different stages of bone formation, including osteogenesis [137]. Furthermore, it has been proposed that miR-92a, miR-16, and let-7a could serve as biomarkers for diagnosing osteogenesis imperfecta since their expression was detected to be altered in serum samples of patients suffering from this disease [138].

One of the primary investigations regarding the roles of miRNAs in osteogenesis imperfecta demonstrated that miR-29b is responsible for the regulation of collagen protein conglomeration during bone mineralization; besides, it was noticed that this miRNA-based regulatory mechanism is dependent on the quantity of *COL1A1* mRNA present in the organism. Nevertheless, the interaction between miR-29b and *COL1A1* has not yet been elucidated. Since *COL1A1* is one of the main genes that encodes collagen 1 (a key component of bone matrix), more studies are needed to ascertain the therapeutic potential of miR-29b and its relation with *COL1A1* [135]. Another similar investigation implied that treatment with ossotide (a mixture that contains organic calcium, amino acids, and growth factors) induced the expression of miR-145 in osteoblasts obtained from osteogenesis imperfecta patients. Consequently, the upregulation of miR-145 elicited the manifestation of proteins associated with osteoblast differentiation (i.e., RUNX2 and OSX) and triggered the Wnt signaling, which is involved in osteoblast proliferation. Despite these discoveries, osteogenesis is a complex process in which several molecular pathways participate [137]; therefore, in-depth knowledge of the phenomenon is required to develop miRNA-based therapeutic strategies that may enhance osteoblast cell differentiation during osteogenesis.

On the other hand, it has been demonstrated that the infusion of MSCs boosts growth in children with osteogenesis imperfecta [139]. Therefore, Otsuru et al. [140] examined the extracellular vesicles produced by MSCs to determine the trophic element that produces the aforesaid therapeutic effect. They found that the extracellular vesicles secreted by MSCs contained miRNAs that may stimulate chondrocyte proliferation; nevertheless, the identification of these miRNAs was not performed during this investigation. Accordingly, more studies are required to accomplish the characterization and functional annotation of these regulatory molecules, which may be helpful to design cell-free treatment for osteogenesis imperfecta in the following years.

### 6.3. Osteomyelitis

Osteomyelitis is a type of musculoskeletal infection induced by pathogenic organisms (e.g., *Staphylococcus aureus*, *Streptococcus pneumoniae*, and *Kingella kingae*) and characterized by the inflammation of bone and bone marrow. Additionally, the development of this disease can lead to necrotic bone, local bone destruction, and the apposition of new bone [141,142]. Since the design of therapeutic strategies for osteomyelitis represents a clinical challenge, the in-depth study of the miRNA-based regulatory mechanisms of its pathogenesis might be a potential source of novel treatments; nevertheless, there is still a limited number of investigations in this arena.

An initial study revealed that miR-24 is downregulated in the blood samples of patients suffering from osteomyelitis, as well as in mouse clonal pre-osteoblastic cells belonging to the cell line MC3T3-E1 that were infected with *S. aureus*. Besides, it was observed that the *S. aureus*-infected MC3T3-E1 cells displayed osteoblast apoptosis, cell proliferation inhibition, and blockage of both bone formation and mineralization [143]. The target of miR-24 is the chitinase 3-like 1 (*CHI3L1*) gene, which is related to the synthesis of a secretory glycoprotein involved in different cancers and inflammatory conditions. Moreover, the overexpression of miR-24 was able to counteract the effects of the *S. aureus* infection in MC3T3-E1 cells by means of increasing cell proliferation, reducing the percentage of apoptotic cells, and affecting the bone formation and differentiation. These results support the idea that miR-24 should be thoroughly studied in the following years as a potential drug target for osteomyelitis [143].

Wu et al. [144] developed an osteomyelitis model treating human BMSCs with the staphylococcal protein A (SpA) during osteogenic differentiation. Afterward, they elucidated that miR-541-3p is negatively modulated by the lncRNA FAM83H-AS1, while Wnt family member 3A (*WNT3A*, a key regulatory factor of osteogenic differentiation) is the target gene of miR-541-3p. Finally, they concluded that the FAM83H-AS1 ameliorated the inhibited osteogenic differentiation produced by the SpA via regulating the miR-541-3p/*WNT3A* pathway. Hence, this molecular mechanism could represent a prominent source of therapeutic approaches for osteomyelitis. Throughout a further investigation, lipopolysaccharide-induced osteomyelitis-like models of MC3T3-E1 cells were treated with a methanolic extract of the alga *Cladophora glomerata* enriched with Mn(II) ions; remarkably, those cultures treated with 1% of the algal extract presented a significant upregulation in the expression of miR-27a, miR-29b, and miR-21-5p, which target specific genes that are crucial for MC3T3-E1 differentiation, such as *APC*, *DKK1*, and *SFRP2*. Additionally, this extract allowed the restoration of bone mineralization, impeded osteoblast apoptosis, and reestablished the expression of osteoblast-specific genes, thus suggesting that this treatment may be considered within the design of therapies against bacteria-induced osteomyelitis [145].

In 2020, Ma et al. [146] reported that in the osteomyelitis patients and *S. aureus*-infected MC3T3-E1 cells, the endothelial nitric oxide synthase (eNOS) was downregulated, whereas tumor necrosis factor-⍺ (TNF-⍺) and miR-129-5p were upregulated. These outcomes show that the upregulation of TNF-⍺ augments the expression of miR-129-5p, which, therefore, is able to decrease the occurrence of eNOS. As a matter of fact, this molecular interaction stimulates the appearance of mineralization defects and contributes to the progress of osteomyelitis. Interestingly, researchers observed that both the inhibition of miR-129-5p and the ectopic expression of eNOS rescued the mineralization defect in *S. aureus*-infected MC3T3-E1 cells. Accordingly, the TNF-⍺/miR-129-5p/eNOS signaling pathway is a molecular mechanism that deserves further examination to understand its impact on the pathology of *S. aureus*-induced osteomyelitis [146].

### 6.4. Multiple Myeloma Bone Disease (MMBD)

Multiple myeloma is a monoclonal plasma cell disorder representing 10% of all hematologic cancers [147]. Multiple myeloma bone disease (MMBD) is a critical clinical manifestation of multiple myeloma characterized by excessive bone osteolysis caused by increased osteoclastogenesis and decreased differentiation of BMSCs into osteoblasts. Moreover, in this type of condition, cancer cells gather within the bone marrow microenvironment, hence displacing healthful blood cells. For this reason, cancer cells produce abnormal antibodies and other proteins instead of producing useful ones, which affect diverse bone structures, such as the skull, ribs, pelvis, and vertebral column [148].

Remarkably, a set of previous studies have proposed that the miRNA-based regulation of gene expression could have a major impact on the progression of this disease. In fact, the altered expression of diverse miRNAs, such as miR-15, miR-16, miR-21, miR-221, and miR-34 family could influence the pathogenesis process of multiple myeloma [149]. In 2011, a research revealed that the overexpression of miR-29b downregulates myeloid-cell leukemia 1 (Mcl-1), which in consequence triggers the apoptosis of multiple myeloma cells [150]. Furthermore, miR-34a encapsulated in chitosan/PLGA nanoparticles inhibited tumor growth in murine xenograft models of human multiple myeloma disease since this miRNA directly downregulates proteins associated with tumor development, i.e., Bcl-2, Notch 1, and CDK6 [151].

Additionally, the circulating miRNAs miR-143, miR-144, miR-199, and miR-203 were detected to be downregulated in multiple myeloma patients, and since these miRNAs might regulate the expression of Versican (a proteoglycan that promotes antigen-presenting cell tolerance in myeloma tumors), they have been proposed as biomarkers for this disease. In particular, researchers detected that miR-203 has a strong association with myeloma-related parameters. This attribute suggests that miR-203 may have a prospective use for the molecular diagnosis of multiple myeloma [152]. Yu et al. [153] reported that the exosomal circular ARN ATP10A could be a useful prognostic biomarker for multiple myeloma since it may enhance angiogenesis via targeting hsa-miR-6804-3p, hs-miR-6758-3p, hsa-miR-3977, hsa-miR-1266-3p, and hsa-miR-3620-3p, therefore modifying the expression of their downstream mRNAs (e.g., PDGF, FGF, VEGFB, and HIF1A).

As observed, the analysis of the miRNA transcriptome in recent years has provided a better understanding of the molecular mechanisms behind multiple myeloma. Most importantly, it has been demonstrated that miRNAs can represent an alternative theragnostic tool for this type of cancer.

### 6.5. Thalassemia Bone Disease (TBD)

Thalassemia, also known as β-thalassemia, is a type of inherited autosomal recessive hemoglobinopathy caused by a single gene mutation. It is mainly characterized by a chronic hemolytic anemia triggered by an incorrect hemoglobin synthesis linked with the absence or reduced production of β-globin chains. The insufficient synthesis of β-globin chains creates an imbalance between the α- and β-, δ- and γ-globin chains that lead to the precipitation of excess α-chains, thus aggravating an ineffective process of erythrocyte production within the bone marrow [154,155,156]. Thalassemia bone disease (TBD) is a common and severe complication of thalassemia. Patients with β-thalassemia are commonly treated with regular blood transfusions, interventions such as stem cell transplantation, iron chelating, and gene therapy. However, these treatments may cause health complications for patients [157].

In previous studies, many miRNAs (e.g., miR-326, let-7, miR-96, miR-144, miR-451, and miR-150) have displayed an important role in thalassemia by elevating or inhibiting α-, β- and γ-globin expression. Therefore, these miRNAs are considered as potential theragnostic targets for thalassemia [157]. Kuno et al. (2019) [158] noticed a significant upregulation of miR-125b in activated phagocytic monocytes of patients with β-thalassemia. These expression levels were linked with the phagocytic activity of the monocytes; in addition, miR-125b may possess a noteworthy role as a genetic modifier in anemia severity in patients with β-thalassemia. Interestingly, it has been observed that β-thalassemia intermedia (TI) patients with higher fetal hemoglobin levels (HbF) can improve their disease condition. In this regard, Gholampour et al. [156] revealed that miR-30a regulates the expression levels of HbF and improves symptoms of anemia in β-thalassemia patients by targeting BCL11A (a zinc finger protein that is required for lymphocyte and erythroid lineage development).

Later, twelve miRNAs were found associated with both the MAPK and HIF-1 signaling pathways, which are implicated in HbF upregulation. Eight of these miRNAs were upregulated (i.e., hsa-miR-146a-5p, hsa-miR-146b-5p, hsa-miR-148b-3p, hsa-miR-155-5p, hsa-miR-192-5p, hsa-miR-335-5p, hsa-miR-7-5p, and hsa-miR-98-5p), while the other four were downregulated (i.e., hsa-let-7a-5p, hsa-miR-320a, hsa-let-7b-5p, and hsa-miR-92a-3p). Besides, researchers observed that the miR-17/92 cluster is crucial for HbF regulation, thus enlightening an opportunity to manipulate these miRNAs to improve HbF levels in β-thalassemia patients [155]. Since increasing HbF levels seems to be a promising treatment for β-thalassemia, recent studies have shown that miR-15a and miR-486-3p have a crucial role in increasing both γ-globin and HbF expression levels, hence reducing blood transfusion dependency [154]. Nevertheless, further studies must be done to validate these findings and pave the way for preclinical and clinical assays of miRNA-based drugs for β-thalassemia.

### 6.6. Clinical Applications of miRNAs in Other Bone Diseases

The findings reported in the last few years elucidate that miRNAs are promising theragnostic targets for those bone diseases whose relationship with the miRNA transcriptome has so far been barely examined, i.e., atrophic non-union, osteogenesis imperfecta, osteomyelitis, multiple myeloma, and thalassemia. As mentioned in previous sections, several challenges must be overcome for miRNA-based drugs to reach the pharmaceutical breakthrough (e.g., developing efficient delivery systems, studies with larger sample sizes and controlled diets, and toxicity analysis). Therefore, future research should be centered on understanding the therapeutic effects and diagnostic potential of miRNAs in the aforesaid group of bone diseases.

Some of the most significant roles of miRNAs during the progression of bone diseases are shown in Table 1.

## 7. Conclusions

Throughout the past years, extensive research has been performed to understand the functional roles of miRNAs within the etiology and progression of numerous bone diseases, such as osteoporosis, osteosarcoma, osteonecrosis, and bone metastasis, among others. In fact, the combination of bioinformatic tools with experimental procedures has allowed scientists to detect dysregulated miRNAs that could work as biomarkers and therapeutic targets for the abovementioned bone diseases. Nonetheless, even though the advances in understanding the interplay between the miRNA transcriptome and bone disease-associated genes are very encouraging, further studies are required to develop novel miRNA based therapeutic drugs overcoming the risks associated with the existing treatments for bone diseases (e.g., the toxicity of long-term doses and the secondary effects of radiotherapy). Likewise, the detection (as well as profiling of expression pattern) of bone disease-associated miRNAs could serve as potential biomarkers for the proper diagnosis of bone diseases and their severity. Therefore, continuing to analyze the roles of miRNAs in the development and progression of bone diseases, especially those barely studied in this research arena, could be very promising.

## 8. Future Prospects

As depicted throughout this review, molecular biologists have been working tirelessly to unveil the miRNA-mediated molecular mechanism underlying bone disease pathophysiology. However, many unsolved questions regarding the genetic etiology of bone diseases deserve further analysis in the forthcoming years, e.g., a wide range of bone disease-associated genes whose miRNA-based regulation has not yet been thoroughly studied; some examples of these genes are *CLCN7*, *WNT1*, *IFITM5*, *SERPINF1*, *CRTAP*, *PSL3*, *COL1A1,* and *COL1A2* [159,160]. In the same sense, diverse investigations have demonstrated the importance of multiple miRNAs in bone development, such as miR-10b, miR-19a-3p, miR-26b, miR-92a, miR-130a, miR-135-5p, and miR-374b [161]; nonetheless, there are few reports about their potential theragnostic roles in bone diseases.

On the other hand, since bone formation is closely related to the vitamin D-mediated mineralization of osteoblasts [162], the therapeutic potential of those miRNAs that regulate this molecular process should be analyzed to find alternative strategies to relieve bone diseases. In this context, it has been reported that miR-125b, miR-27b, miR-21, miR-181, miR-326, among other miRNAs, have an important functional implication within vitamin D production, metabolism, and signaling pathways [163]. Moreover, to the best of our knowledge, information about the sex-dependent miRNA expression in bone diseases is limited; few studies that are linked with this topic indicated that the expression of most of the circulating miRNAs in osteoporosis patients is sex-independent [164,165]; however, more investigations are required to confirm the non-sex-biased expression of miRNAs during the development and progression of bone disorders.

Remarkably, an interesting ncRNA-based mechanism that may have a potential therapeutic role in bone diseases is the sponging effect of lncRNAs against miRNAs [166]. The LEF1-AS1/miR-24-3p, MCF2L-AS1/miR-33a, and MALAT1/miR-30 are some examples of these interactions that occur during osteogenesis [167]. Other intriguing lncRNA-miRNA interactions with promising clinical roles that were discussed within this review are MSC-AS1/miR-140-5p (osteoporosis) [59], SNHG16/miR-205 (osteosarcoma) [79], and FAM83H-AS1/miR-541-3p (osteomyelitis) [144]. Nevertheless, more lncRNAs -miRNAs-based research is needed to develop novel therapeutic strategies against bone diseases. In addition, data regarding the implications of the miRNA transcriptome in other less common metabolic bone diseases, such as rickets, fluorosis, hyperparathyroidism, hypophosphatasia, sclerosteosis, and tumor-induced osteomalacia [160,168], are still scarce and hence need to be studied.

Last but not least, given that the FDA approved the use of small RNA drugs in clinical medicine, investigations regarding the preclinical and clinical outcomes of miRNA-based therapeutics have increased significantly in the last few years. As a matter of fact, miRNAs and anti-miR oligonucleotides are proposed as potential drugs for a number of multifactorial diseases with no effective therapies [169]. Consequently, this research arena is gaining more attention from investors, and several pharmaceutical companies have been created to design miRNA-based drugs (e.g., miRagen Therapeutics Inc., Mirna Therapeutics Inc., and SantarisPharma) [10]. Under this premise, some of the most promising medications based on miRNA technology that have been entered into clinical trials are miravirsen and RG-101 (anti-miRs for hepatitis C) [170,171], MRX34 (miRNA mimic for cancer therapy) [172], RG-012 (anti-miR for Alport syndrome) [173], and RGLS4326 (anti-miR for polycystic kidney disease) [174].

In addition, various miRNA-based drugs, including MGN-1374 (anti-miR for post-myocardial infarction treatment) [175], MGN-2677 (anti-miR for vascular disease) [176], and MGN-4220 (anti-miR for cardiac fibrosis) [177], amongst others, are currently at preclinical stages. Nevertheless, since the research on the functional implications of miRNAs in bone diseases is still at an early stage, no miRNA-based drugs for these ailments are entered yet into the clinical trial. However, we believe that the information presented in this current review will reinforce the study of the association between the miRNA transcriptome and bone diseases to establish the drug pipeline for clinical trials.

## Figures and Tables

**Table 1:** Functional implications of miRNAs in the development and progression of bone diseases.

Bone Disease	miRNA	miRNA Regulation	Target	Biological Implication	Reference
Osteoporosis	miR-148a	Upregulated	ER-α	Inhibition of osteoblast cell growth and osteoblast apoptosis	[49]
	miR-122-5p	Downregulated	ER-α	Development of osteoporosis	[50]
	miR-144-3p	Downregulated	RANK	Osteoclastogenesis alteration	[51]
	miR-133a	Upregulated	RUNX2	Osteoclast differentiation and loss of bone density	[52]
	miR-363-3p	Upregulated	PTEN	Osteoclastogenesis promotion and inhibition of osteogenic differentiation	[41]
	miR-29a	Downregulated	RANKL	Osteoclastogenic differentiation	[55]
	miR-152	Upregulated	RICTOR	Inhibition of osteoblast differentiation	[35]
	miR-579-3p	Upregulated	SIRT1	Inhibition of osteogenic differentiation	[57]
	miR-200a-3p	Upregulated	GLS	Inhibition of osteogenic differentiation	[58]
	miR-140-5p	Downregulated	BMP2	Enhancement of osteogenic differentiation	[59]
	miR-339	Downregulated	*DLX5*	Enhancement of osteogenic differentiation	[60]
	miR-140-3p	Upregulated	*MCF2L*	Inhibition of preosteoblast viability and induction of preosteoblast apoptosis	[61]
	miR-194-5p	Upregulated	WNT5A	Inhibition of bone formation and osteoblast/osteogenic differentiation	[62]
	miR-1286	Upregulated	FZD4	Inhibition of osteogenic differentiation	[63]
	miR-483-5p	Upregulated	IGF2	Promotion of osteoclast differentiation	[34]
	miR-203a	Upregulated	*DLX5*	Osteogenic differentiation delay and bone loss	[40]
	miR-10a-3p	Downregulated (by a kaempferol treatment)	CXCL12	Promotion of osteogenic differentiation	[64]
	miR-300	Downregulated	*CUL4B*	Degradation of PTEN (tumor suppressor)	[71]
	miR-93	Upregulated	P21	Proliferation of osteosarcoma cells	[72]
	miR-411	Upregulated	MTSS1	Osteosarcoma cell migration and proliferation	[73]
	miR-1284	Downregulated	HMGB1	Osteosarcoma cell migration and proliferation	[76]
	let-7a	Downregulated	E2F2	Osteosarcoma development	[77]
	miR-1301	Downregulated	BCL9	Cell proliferation, invasion, and migration	[81]
	miR-487a	Upregulated	-	-	[82]
	miR-493-5p	Upregulated	-	-	
	miR-501-3p	Upregulated	-	-	
	miR-502-5p	Upregulated	-	-	
	hsa-miR-19-3p	Upregulated	Several transcription factors	Cell proliferation and carcinogenesis	[83]
	hsa-miR-106b-3p	Upregulated			
	hsa-miR-543	Downregulated	*ZNRD1*	Osteosarcoma chemoresistance	[84]
Osteonecrosis	hsa-miR-195-5p	Downregulated	157 different genes	Osteoblast dissemination disruption, accelerated cell apoptosis, and collapse of the femoral head	[94]
	hsa-miR-601	Upregulated	238 different genes	Adipogenic and osteogenic differentiation	[87]
	hsa-miR-452-3p	Upregulated		Adipogenic and osteogenic differentiation	
	hsa-miR-647	Upregulated		Adipogenic and osteogenic differentiation	
	hsa-miR-516b-5p	Upregulated		Adipogenic and osteogenic differentiation	
	hsa-miR-127-5p	Upregulated		Adipogenic and osteogenic differentiation	
	hsa-miR-122-3p	Downregulated		Adipogenic and osteogenic differentiation	
	miR-181d	Upregulated	*SMAD3*	Inhibition of osteogenic differentiation	[88]
	miR-217	Downregulated	*DKK1*	Inhibition of cell proliferation and osteogenic differentiation	[96]
	miR-214	Upregulated	ATF4 and PTEN	Inhibition of osteoblast differentiation and promotion of osteoclast function	[97]
	miR-186-5p	Upregulated	CXCL13	Alteration of cell viability and osteoblastic differentiation	[89]
	miR-410	Downregulated	Wnt-11	High levels of osteoclasts and low levels of osteoblasts. Low bone mineral density	[98]
	miR-93-5p	Upregulated	-	-	[86]
	miR-320a	Upregulated	-	-	
	hsa-miR-378-c	Upregulated	WNT3A, DACT1 and CSF1	Bone remodeling and angiogenesis during ONFH	[99]
	hsa-let-7a-5p	Upregulated	RCAN2 and IL9R	Progression of ONFH	
	hsa-miR-3200-5p	Upregulated	RELN	Progression of ONFH	
	hsa-miR-28-5p	Upregulated	RELA	Cartilage degeneration	
	hsa-miR-532-5p	Upregulated	CLDN18 and CLDN10	Bone loss	
Bone Metastasis	miR-466	Downregulated	RUNX2	Inhibition of apoptosis and cell cycle arrest. Cell migration, proliferation, and invasion	[109]
	miR-19a-3p	Downregulated	*SMAD2* and *SMAD4*	Cell migration, invasion, and bone metastasis	[110]
	miR-582-3p	Downregulated	SMAD2, SMAD4, and TGFBR1	Cell migration, invasion, and metastasis	[111]
	miR-582-5p	Downregulated	SMAD2, TGFBR1 and TGFBR2		
	miR-96	Upregulated	E-Cadherin and EpCAM	Cancer cell metastasis within bone microenvironment and tumor development	[113]
	miR-214-3p	Upregulated	*TRAF3*	Osteolytic bone metastasis and elevated bone resorption	[116]
	miR-124	Downregulated	IL-11	Bone metastasis of breast cancer cells	[117]
	miR-139-5p	Downregulated	NOTCH1	Osteogenic differentiation and lytic bone disease in lung cancer	[118]
	miR-34a	Downregulated	C-IAP2 and Bcl-2	Tumor invasion and metastasis	[120]
	hsa-miR-940	Upregulated	ARHGAP1 and FAM134A	Osteogenic differentiation and induction of osteoblastic lesions in tumors	[121]
Atrophic non-union	miR-31a-3p	Upregulated	FGF3	Osteogenesis, chondrogenesis, and impairment of fracture healing	[131]
	miR-31a-5p	Upregulated	SATB2, Osterix, RUNX2, BMPR2, and NIK	Osteogenic differentiation	
	miR-146a-5p	Upregulated	TRAF6, IRAK1, CXCR4, and SDF-1	Development of non-union	
	miR-146b-5p	Upregulated	TRAF6 and IRAK1	Development of non-union	
	miR-223-3p	Upregulated	STAT3 and IGF1R	Development of non-union	
	miR-628-3p	Upregulated	RUNX2	Inhibition of osteoblast differentiation	[129]
	miR-381	Upregulated	*WNT5A* and *FZD3*	Inhibition of osteogenic differentiation	[132]
	miR-1323	Upregulated	BMP4 and SMAD4	Inhibition of osteogenic differentiation and development of atrophic non-union	[133]
	hsa-miR-149*	Upregulated	*ALPL*	Development of atrophic non-union	[134]
	hsa-miR-221	Upregulated	*PDGFA*	Development of atrophic non-union	
	hsa-miR-654-5p	Upregulated	*BMP2*	Development of atrophic non-union	
Osteogenesis imperfecta	miR-29b	Downregulated	-	Altered regulation of collagen protein accumulation	[135]
	miR-145	Upregulated (by an ossotide treatment)	RUNX2 and OSX	Enhancement of osteoblast cell differentiation and proliferation	[137]
Osteomyelitis	miR-24	Downregulated	*CHI3L1*	Inhibition of cell proliferation, blockage of both bone formation and mineralization, and osteoblast apoptosis	[143]
	miR-129-5p	Upregulated	eNOS	Occurrence of mineralization defect and progression of osteomyelitis	[146]
Multiple myeloma	miR-29b	Downregulated	Mcl-1	Survival ofmyeloma cells	[150]
	miR-143	Downregulated	Versican	Myeloma-associated parameters	[152]
	miR-144	Downregulated			
	miR-199	Downregulated			
	miR-203	Downregulated			
Thalassemia	miR-30a	Upregulated	BCL11A	Increased expression levels of HbF and a decreased expression levels of ferritin	[156]
	miR-15a	Upregulated	MAF proteins and *MYB*	HbF induction	[154]
	miR-486-3p	Upregulated	MAFK, BCL11A, MTA1, and NR2F2		

## Data Availability

The datasets generated during and/or analyzed during the current study are available from the corresponding author on reasonable request.
